# Hair care and epidemiological‐clinical profile of traction alopecia among women in hair salons in Yaoundé, Cameroon

**DOI:** 10.1002/ski2.158

**Published:** 2022-08-24

**Authors:** Letitia Fiona Mbussuh Nzeng, Georges Nguefack‐Tsague, Rose Kotto, Dahlia Noëlle Tounouga, Odette Berline Sigha, Grace Anita Nkoro, Martine Nida, Emmanuel Armand Kouotou

**Affiliations:** ^1^ Faculty of Medicine and Biomedical Sciences Université of Yaoundé 1 Yaounde Cameroon; ^2^ Faculty of Medicine and Pharmaceutic Sciences Université of Douala Douala Cameroon; ^3^ National University Teaching Hospital of Cotonou Cotonou Benin; ^4^ Faculty of Health Sciences University of Bamenda Bambili Cameroon

## Abstract

**Background:**

Hair is valued by all individuals especially women. The perception of beauty is specific to everyone. In order to fulfil their ideal beauty, women use various methods to modify their hair's appearance. Afro hair is particularly fragile, so using these methods can be particularly deleterious for black women's hair.

**Objectives:**

Describe hair care practices of women living in Yaoundé, determine the prevalence of traction alopecia (TA) and describe its clinical profile.

**Methods:**

We carried out a cross‐sectional study in hairdressing saloon in Yaoundé. A questionnaire was administered and scalp exams were performed to determine Marginal TA severity score.

**Results:**

We included 223 women with a mean age of 24.9 ± 7 years. The prevalence of TA was 34.5%. Mild and moderate stages were most represented. As far as haircare is concerned, extensions were regularly used by 95.1% of participants. Wigs were regularly worn by 58.7% of participants. Chemical hair straightening was done by 87.9% of women and was performed twice to thrice a year by 43.9%. Almost 76% of women used hair straightener and hair dryer. Hair washing was done monthly by 43.8% of participants and the main cosmetic used was shampoo (75.3%).

**Conclusion:**

Traction alopecia is a very common disease in women living in Yaoundé. Extensions, wigs and shampooing are their main hair care practices.

## Graphical abstract

Traction alopecia is a very common disease in women living in Yaoundé up to 34.5% of the studied population is affected. Extensions, wigs and shampooing are their main hair care practices.

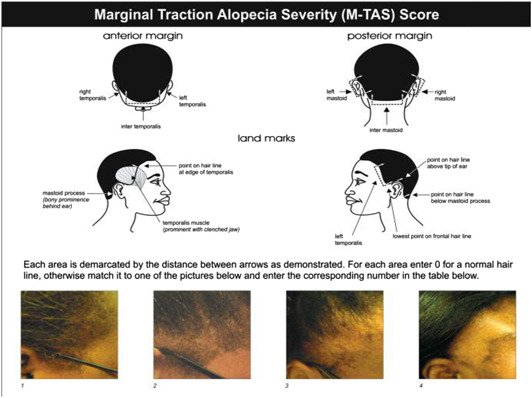

1

**What is already known about this topic?**

- Traction alopecia (TA) is a common condition in Africa and particularly in Cameroon, but there is little studied.

**What does this study add?**

- We have assessed here the prevalence and clinical profile of TA in Cameroon as well as the different hair treatments used.

## 2 BACKGROUND

Hair is a part of individual identity of men and women.^1^, ^2^, ^3^ They are both cherished and desired in all societies.^1^ In women in particular, hair is an element of her charm. Also, hair represents a changeable physical trait in terms of length, colour or shape. In addition, each individual has their own perception of beauty, thus justifying the usage of various cosmetics and hairstyles for beauty purposes.^2^

Hair characteristics vary from one individual to another. The most widely used hair classification distinguishes three types of hair: African (Negroid), Caucasian and Asian.^4^ African hair is relatively fragile due to its unique characteristics.^5^ Indeed, African hair has reduced elasticity, is less resistant and tangles more easily than other types of hair.^4^ Moreover, it is dry and has the lowest growth rate.^6^

These specificities make the daily maintenance of African hair difficult and could justify the dissatisfaction of black women with their hair in its natural state.^7^, ^8^ Therefore, the desire to fulfil aesthetic norms of global fashion might influence the choice of hair care among black women.^4^, ^9^

However, usage of cosmetics is not without risk. Indeed, some cosmetics and hair care products could cause significant hair damage, including alopecia.^4^, ^7^, ^10^ Alopecia is a common reason for consultation in dermatology.^11^ Alopecia can be traumatic or non‐traumatic, cicatricial or non‐cicatricial, localized or diffuse. Traction Alopecia (TA) in particular, is a type of non‐cicatricial and localized traumatic alopecia, which is very often found in black subjects due to their cosmetic habits (thick hairstyles; hair straightening, hair drying, flat irons etc.).^12^

In Africa, TA is a common condition that remains poorly understood and less accepted culturally.^13^ In worst case, TA can progress to permanent hair loss.^6^ Moreover, this condition would induce a significant psychological impact, in particular because of aesthetic damage it causes.^3^, ^14^, ^15^

In Cameroon, a black African country, there are practically no studies that have assessed the prevalence of TA in the general population, and specifically within women. So, this study aimed to determine the prevalence and clinical profile of TA in a group of women, as well as to describe the different hair treatments used.

## 3 METHODOLOGY

### 3.1 Design and location of the study

We carried out a descriptive cross‐sectional study conducted for June to July 2020 in 29 hair salons (23 classic salons and 6 VIP salons) in Yaoundé, Cameroon. As we didn't have the exact number of hair salons in the city, salons were randomly selected in each district of Yaoundé. A classic hair salon/VIP hair salon ratio has not been defined.

The sample size was 171 participants and given by the following formula:

$$n=\frac{t^{2}\times \;p\times \left(1-p\right)}{m^{2}}$$

*n:* minimum sample size for statistically significant results;

*t:* 1.96 for a confidence interval of 95%;

*p:* prevalence of traction alopecia in women 31.2%;

*m* ^2^: the absolute risk of error 0.07.

Our target population was women living in Yaoundé, Cameroon and our source population was women visiting hair salons in the city. Women aged at least 18 presents in the hairdressing salon during our visit and having given their free and informed consent were included in our study. Women who didn't complete the interview, women who have not had their hair done for at least 5 years and those wearing hairstyles covering all the scalp were excluded.

### 3.2 Data collection procedure

We have obtained an ethical clearance from the institutional ethics and research committee of the Faculty of Medicine and Biomedical Sciences of the University of Yaoundé I. Subsequently, authorisations were given by the administrative authorities of the various districts of Yaoundé as well as by the owners of the various hair salons selected for the study.

In the hair salon, the purpose of the study was presented to each potential participant and their consent to take part was required. Then, the pre‐designed and pre‐tested data collection tool was completed under the supervision and help of the interviewer. The questionnaire contained socio‐demographic information (age, sex, occupation, income, ethnic) as well as the various hair care treatments practiced by the participant (hairdressing habits, practice of hair straightening, hair hygiene). The investigator checked and ensured the completeness of the information. Subsequently, a scalp examination was carried out in the hairdressing salon, at a space dedicated to this act. To do this, the examiner inspected the scalp of the participants, and appreciated the hair density. The scalp margins were divided into anterior and posterior margins by an imaginary line joining the two tragus.

**FIGURE 1:**
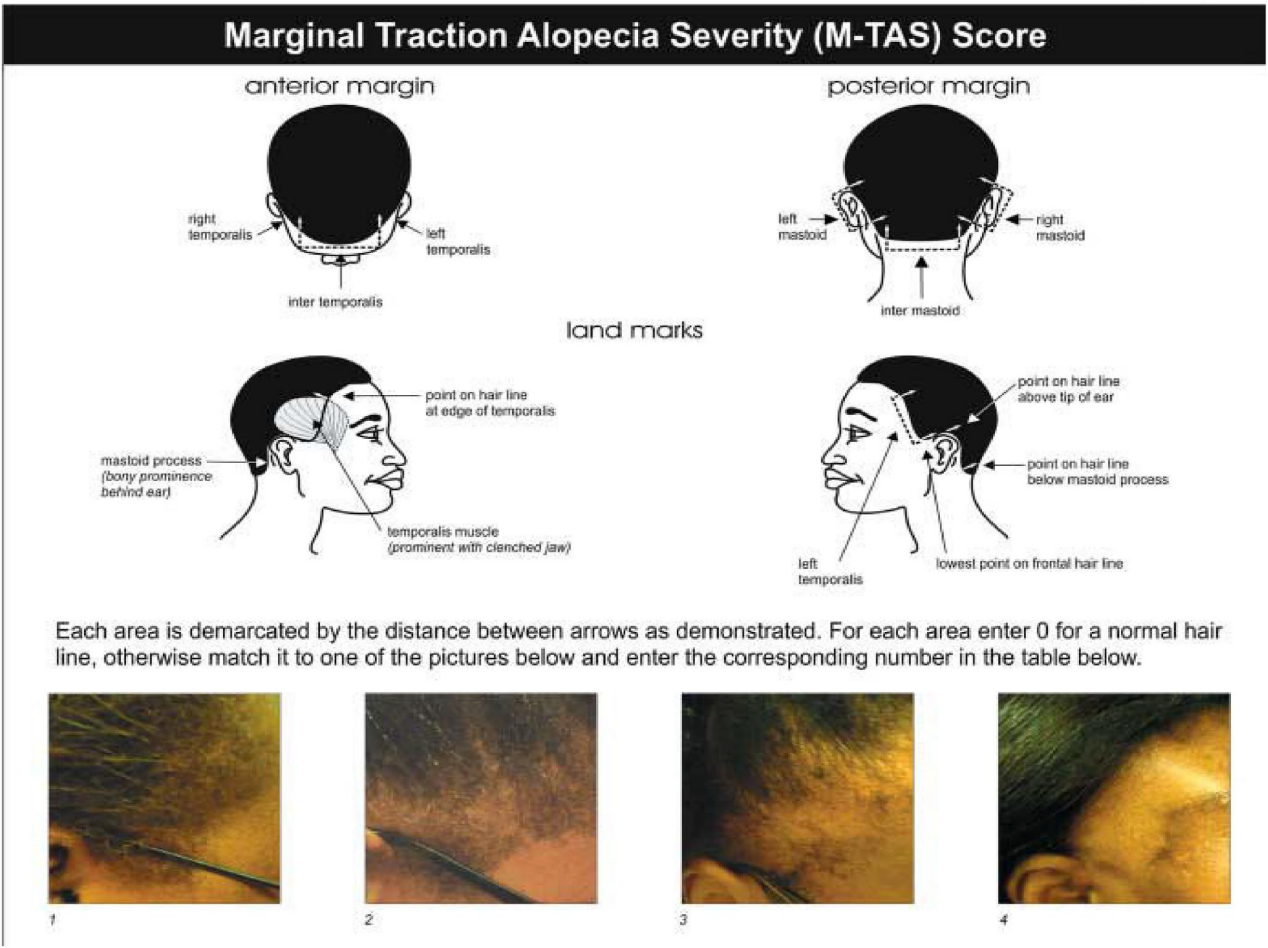
Image grid for the evaluation of the Marginal Traction Alopecia Score

As a reminder, the medial edges of the temporal muscles divide the anterior margin into three regions: left temporal, right temporal and intertemporal. The temporal muscles were palpated by asking the participant to “clench their teeth”. The posterior margin is also subdivided into three regions: left mastoid, right mastoid and intermastoid by the two mastoid processes.^16^ The Marginal Traction Alopecia Severity Score (M‐TAS) image grid proposed by Khumalo et al. (Figure 1) was used to evaluate TA.^17^ When the examination of a region was normal, the score 0 was assigned meaning the absence of traction alopecia. On the other hand, when areas of alopecia or thinning hair were detected, the severity was assessed using the ‘image grid’ of the questionnaire with the aim of assigning a score varying between 1 and 4. The scores for each region were added together to obtain a total. The M‐TAS ranges from 0 to 24 and interpreted as follows^16^:

- M‐TAS = 0: no traction alopecia
- 1 ≤ M‐TAS ≤ 3: mild traction alopecia
- 4 ≤ M‐TAS ≤ 6: moderate traction alopecia
- M‐TAS ˃ 6: severe traction alopecia

At the end of this process, counselling was given to all participants on TA and methods of prevention. Similarly, counselling was done on changing behaviours related to hygiene and hair care.

### 3.3 Data analysis

The data was collected using Epi Info software version 7.2.5 and analyzed with IBM SPSS software version 25 for Windows. Quantitative variables were expressed by mean ± SD when their distribution followed the normal distribution; Otherwise, they were expressed by median ± IQ. Qualitative variables were expressed by numbers and percentages. Prevalence was expressed by his 95% confidence intervals (CI).

### 3.4 Ethical and administrative considerations

We conducted our study in strict accordance with the fundamental principles of the Helsinki Declaration on Research Involving Persons. The aspects and procedures were fully presented to each potential participant and we included only those who voluntarily gave their consent to take part. Those who refused to participate to the study did not suffer of any prejudice. In addition, our research protocol had previously been submitted to the institutional ethics and research committee of the Faculty of Medicine and Biomedical Sciences of the University of Yaoundé I. Authorisations were also obtained from the various authorities.

## 4 RESULTS

**FIGURE 2:**
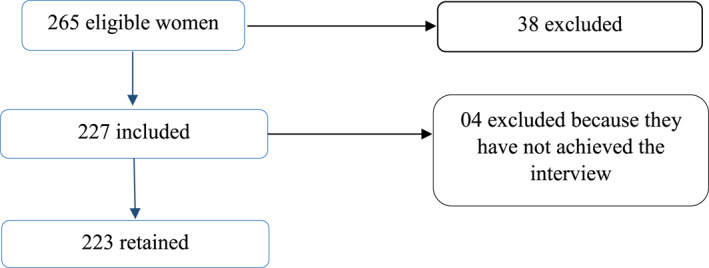
Sample flowchart

We recruited 265 women for the study. Thirty‐eight of them were not included because they did not give their consent. Of the 227 women included, four of them did not complete the interview for various reasons. We therefore retained a total of 223 participants in our study (Figure 2).

The mean age of our sample was 24.9 ± 7 years with range of 18–55 years. More than half of participants (55.1%) were aged between 18 and 24 years old. Students represented 59.2% of our sample. Monthly income was less than XAF 36 000 (65USD) for 63.7% of participants (Table 1).

**TABLE 1:** Distribution of participants according to socio‐demographic characteristics

Characteristics *N* = 223	*n* (%)
Age groups (years)	
[18–24]	123 (55.1)
[25–34]	72 (32.3)
[35–55]	28 (12.6)
Occupation	
Public sector	13 (5.8)
Private sector	29 (13)
Trader	10 (4.5)
Hair dresser	16 (7.2)
Student	132 (59.2)
Unemployed	23 (10.3)
Monthly income	
Less than 36 000 XAF	142 (63.7)
36 000–100 000 XAF	53 (23.8)
More than 100 000 XAF	28 (12.5)

### 4.1 Prevalence of traction alopecia

**FIGURE 3:**
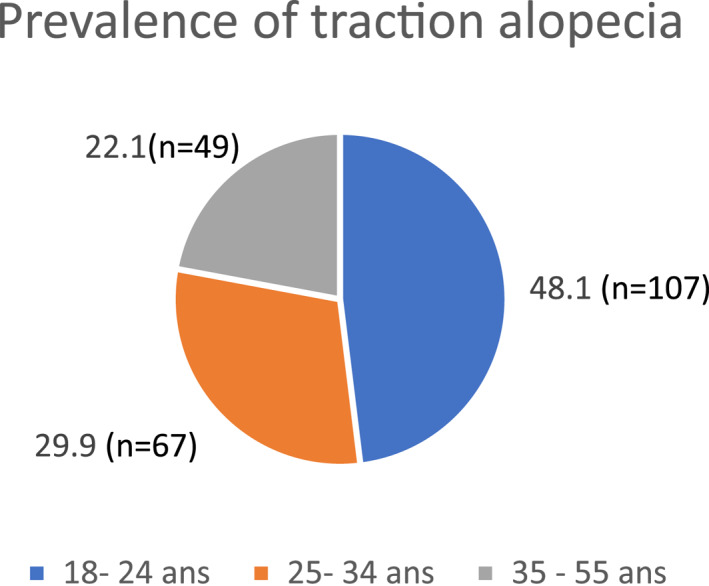
Distribution of traction alopecia according to age groups

Among the 223 women finally included, 77 had TA, meaning a prevalence of 34.5%; (95% CI) = (28.3–40.7). Women under 35 were the most affected (Figure 3).

## 5 HAIR CARE

### 5.1 Hair styling habits

Hairstyles with extensions were worn regularly by 95.1% of participants. The hairstyles were renewed monthly by 63.7% of participants. Also, 26.9% of women wore wigs at least three times a week. Only 13.0% of women did not use beauty accessories such as hats, nets, scarves or caps (Table 2).

**TABLE 2:** Hair styles habits

Characteristics *N* = 223	*n* (%)
Extension	
Yes	212 (95.1)
No	11 (4.9)
Duration of hairstyle	
<3 weeks	81 (36.3)
≥3 weeks	142 (63.7)
Service provider	
Hair dresser	183 (82.1)
Other	23 (10.3)
Both	17 (7.6)
Wearing wigs	
Yes	131 (58.7)
No	92 (41.3)
Frequency of wigs wearing	
<3 times/week	163 (73.1)
≥3 times/week	60 (26.9)
Symptoms after hairstyles	
Always	20 (9)
Sometimes	79 (35.4)
Never/rarely	124 (55.6)
Brushing old braids	
Yes	131 (58.7)
No	92 (41.2)
Traction accessory	
No	29 (13)
Yes	194 (87)

### 5.2 Chemical and heat treatment of hair

As for chemical hair treatment, almost all (87.9%) women had straightened their hair at least once in their lifetime. Nevertheless, it had been carried out by 16.1% of participants before the age of 10 years. Nearly half participants (43.9%) straightened their hair an average of two to three times a year. Burns after straightening were rare or absent in 55.2% of cases (Table 3).

**TABLE 3:** Usage of chemical treatment among participants

Characteristics *N* = 223	*n* (%)
Straightening	
Yes	196 (87.9)
No	27 (12.1)
Age at first straightening	
Less than 10 years	36 (16.1)
More than 10 years	187 (83.9)
Annual frequency of hair straightening	
Just 1 time	75 (33.6)
2–3 times	98 (43.9)
More than 3 times	50 (22.5)
Person doing hair straightening	
Hairdresser	142 (63.7)
Family member	36 (16.1)
No straightening	27 (12.1)
Participant	18 (8.1)
Frequency of burns	
Never/rarely	123 (55.2)
Often	75 (33.6)
Very often/Always	25 (11.2)

36.8% of women had already had coloured their hair. Heat treatment of the hair (hair straightener, hair dryer) was used by 75.8% of participants (Table 4).

**TABLE 4:** Use of heat treatment

Characteristics *N* = 223	*n* (%)
Dyeing	
Yes	82 (36.8)
No	141 (63.2)
Dye <2 weeks after straightening	
Yes	42 (18.8)
No	181 (81.2)
Heat treatment	
Yes	169 (75.8)
No	54 (24.2)

### 5.3 Hair care

More than half (53.8%) women in our sample washed their hair after 3 weeks. Shampoo was the product used by 75.3% of participants. When washing the hair, only 13% did not have traumatic gestures for the hair. Among our participants, 45.3% associated natural oils with industrial oils (Table 5).

**TABLE 5:** Hair hygiene

Characteristics *N* = 223	*n* (%)
Hair washing frequency	
Once a month	120 (53.8)
More than twice monthly	103 (46.2)
Hair washing product	
Shampoo	168 (75.3)
Other	55 (24.7)
Traumatic practices during washing	
No	29 (13)
Yes	194 (87)
Hair oil	
Natural oil	68 (30.5)
Industrial oil	49 (22)
Both	101 (45.3)
None	5 (2.2)

### 5.4 Clinical profile of traction alopecia

The most represented stages of TA were mild and moderate TA which represented respectively 14.3% and 13.5% of sample. Severe TA was found in only 6.7% of participants.

## 6 DISCUSSION

We found that our study population was young. The prevalence of TA was 34.5% and women under 35 were the most affected. 95.1% of participants used extensions for their hairstyles. Wigs were used by 27% of participants. Hair straightening was the most used chemical treatment (87.9%) and women also used heat treatment of the hair (75.8%). Hair washing was done monthly by 53.8% of participants and shampoo was mainly used (73.5%).

The mean age of our study population was 24.9 ± 7 years. Women who visit hair salons in Yaoundé, Cameroon are young. This is the proof of the interest and value that young people attach to the care of their hair. Indeed, the hair is a beauty criterion of woman. They are also associated with woman's fertility and constitute an identity marker.^14^

Prevalence of TA was 34.5% in our sample. This prevalence is similar to that found by Said et al. in 2020 in Egypt which was 36.5%.^18^ On the other hand, our prevalence was much higher than 18% found by Vañó‐Galván et al. in South Africa in 2017.^19^ This difference could be explained by the fact that Vañó‐Galván's included both men and women. However, traction alopecia remains an uncommon entity in men.^13^

The most affected women were under 35 years old. Sayed et al. also found that subjects under age of 45 were the most affected by traction alopecia.^18^ This result could suggest that young women are more at risk of developing traction alopecia during their lifetime.

We found that 95.1% of participants used extensions for their hairstyles. Indeed, hairstyles with extensions are more long lasting and facilitate daily hair maintenance.^20^ Hairstyles were renewed after at least 3 weeks by 63.5% of women in our sample. This result could be explained by the low income of women who prefer more economical hairstyles.

Nearly 27% women in our sample wore wigs at least three times a week. Indeed, wigs are hairpieces used for medical or non‐medical purposes. In dermatology, they are used to minimise the psychological impact of alopecia.^21^ Wigs are an excellent means of camouflage for women with alopecia of any kind. They improve quality of life of these women and contribute to their socialization.^22^ They are easy to maintain, durable and their daily use is not very restrictive.^23^

Up to 87% of our sample used beauty accessories (scarf, cap, bonnet, net). Indeed, women use these accessories very often to protect their hairstyles. Accessories such as scarves have an important cultural value in our society.

Straightening was the main chemical treatment used by our participants (87.9%). All the patients who presented with TA had had to straighten their hair at least once during their life. Chemical hair straightening is a widespread practice among black women probably due to the valuation of straight hair in detriment of frizzy hair.^8^, ^9^, ^13^ Many women used this practice to facilitate hairstyles, to improve self‐esteem and allow their acceptance in society.^13^, ^24^ Also, chemical hair straightening offers more hair styling possibilities.^25^, ^26^ However, hair straightening has been labelled as one of the factors involved in the onset of TA.^27^

Nearly half participants (43.9%) straightened their hair twice or thrice a year. Our results are supported by the work of Dadzie et al. who found an average of two straightening per 88 days.^27^ 44.8% often/always had burns after straightening. This result could indicate non‐compliance or ignorance of hair straightening instructions by providers of this service. Traditional relaxers are emulsions of caustic agents (sodium hydroxide, potassium hydroxide, lithium hydroxide). Therefore, any contact of these products with the skin could lead to burns or irritation.^4^, ^10^

Usage of dyes by women in our study was relatively frequent. More than a third of participants (36.8%) had already coloured their hair at least once in their life. This finding is similar to those of developed countries where nearly 40% of women use hair dyes.^28^

Heat treatment of the hair (hair straightener, hair dryer) was used by 75.8% of participants. The hair dryer is an accessory more and more used in hair salons. It allows rapid drying of the hair and therefore reduces the time required to achieve hairstyles. Usage of hair straightener allows women to give a precise shape to their hair which has been previously straightened.^12^

Studies have shown that TA is more common among women whose hairstyles exerted traction on hair that has previously undergone chemical and/or heat treatment; particularly on straightened hair.^5^, ^29^

In terms of hair hygiene, the proportion of women in our sample washing their hair every 3 weeks (63.7%) was similar to that of women renewing their hairstyles at the same frequency. This would suggest that hair washing was done when renewing the hairstyles in order to ensure the longevity of these hairstyles.^12^ Indeed, it is recommended to shampoo only once a week or every 10 days. Washing daily or several times a week would further weaken frizzy hair. They break easily when they are more frequently and carelessly handled.^30^

Shampoo was the product used by 75.3% of participants. Shampoo is one of the most popular hair care products.^31^ Nowadays, in addition to its primary function which is to wash the hair, shampoos are endowed with revitalising properties and beautify the hair.^32^ Nearly 30.5% of women used natural oils and 45.3% associated natural oils with industrial oils. This result could mean a gradual return to the usage of natural oils. With the advent of the ‘nappy’ movement in recent years, virtues of natural oils on hair are increasingly presented. This would arouse the enthusiasm of women for these oils.

The most represented stages in our sample were mild and moderate TA. Our observation is similar to that of Ngwanya et al. who found mild to moderate TA in almost all participants in her study.^33^ This could mean that traction alopecia progresses gradually from initial stages to terminal stage. In addition, this finding raises the possibility of early diagnosis of this condition for more effective management.^33^

Beyond early diagnosis, TA remains a preventable but understudied pathology. Other studies on a larger scale should be conducted to better understand this pathology and to have figures that could better reflect reality. To date, prevention of TA is essentially based on raising awareness and avoiding risk factors. To this end, public authorities could sensitise women through promotion and initiation campaigns for the maintenance of frizzy hair. Also, slogans and educational messages concerning hair care and TA can be broadcast to parents, children, adolescents and young adults.^29^, ^34^ Similarly, hairdressers should be made aware of compliance with instructions on the use of chemical and heat treatments. As for women, they should choose hairstyles that put little or no tension on the hair shaft, limit the use of extensions as well as usage of thermal and chemical hair treatments.

### 6.1 Limitations of the study

Our study could have limitations, including the fact that: (i) the size of our sample is relatively small due to the short period of data collection; (ii) our study could present an information bias given that some information collected related to previous exposures; (iii) the study was conducted in only one city in the country and therefore these results cannot be generalized to the whole country; (iv) the study took place in hair salons and the prevalence could be overestimated compared to the general population; (v) some data collected may be subjective; (vi) As the study took place in hair salons, the sample obtained could not be representative of the whole population. Nevertheless, this study constitutes a preliminary for the future works on a larger scale of this subject.

## 7 CONCLUSION

Traction alopecia is frequent in our context. Mild and moderate stages are most common. Hair care among women in Yaoundé, Cameroon is dominated by usage of extensions and straightening. Straightening remains the main chemical treatment practiced. Hair straighteners and hair dryers are widely used on a daily basis. Many women had traumatic gestures when washing their hair. Prevention of TA lies in raising awareness and educating populations to avoid risk factors.

## AUTHOR CONTRIBUTIONS

**Leticia Fiona Mbussuh Nzeng**: Conceptualization (supporting); Investigation (equal); Methodology (equal); Writing – original draft (equal). **Georges Nguefack‐Tsague**: Formal analysis (equal); Methodology (equal); Writing – original draft (equal). **Rose Kotto**: Methodology (equal); Validation (equal); Writing – original draft (equal). **Dahlia Noelle Tounouga**: Formal analysis (equal); Writing – original draft (equal); Writing – review & editing (equal). **Odette Berline Sigha**: Methodology (equal); Validation (equal); Writing – original draft (equal). **Grace Anita Nkoro**: Investigation (equal); Methodology (equal); Writing – original draft (equal). **Martine Nida**: Methodology (equal); Validation (equal); Writing – original draft (equal). **Emmanuel Armand Kouotou**: Conceptualization (equal); Methodology (Equal); Supervision (equal); Validation (equal); Writing – original draft (equal)

## CONFLICTS OF INTEREST

The authors confirm that this article content has no conflict of interest.

## ETHICS STATEMENT

Approved by the institutional ethics and research committee of the Faculty of Medicine and Biomedical Sciences of the University of Yaoundé I (AUTORISATION N*2021/045/FMBS/PR/CIE).

## Data Availability

All data relevant to the study are included in the article or uploaded as online supplemental information. No additional data available, all data relevant to the study are included in the article.
